# The complete chloroplast genome of *Agrimonia pilosa* var. nepalensis (D. Don) Nakai

**DOI:** 10.1080/23802359.2021.1888337

**Published:** 2021-03-16

**Authors:** Li Yang, Yun Teng, Shou-Fu Gong, Zhi-Min Feng, Yan Zhang

**Affiliations:** College of Horticulture, Xinyang Agriculture and Forestry University, Xinyang, Henan, China

**Keywords:** Rosaceae, *Agrimonia*, chloroplast genome, plastid genome, phylogeny

## Abstract

*Agrimonia pilosa* var. nepalensis (D. Don) Nakai is an herbaceous species of Rosaceae distributed in China. It has ornamental and ecological values. Lack of genetic background seriously hinders its further research and utilization. To provide genetic information for further study of it, complete chloroplast (cp) genome was characterized in this study. The genome is a circular molecule of 155,147 bp in length with overall GC content of 36.9%, which contains 85 protein-coding genes, eight ribosomal RNA genes, and 37 transfer RNA genes. It contains a typical tetrad structure, including a large single copy, a small single copy, and two inverted repeat regions. Phylogenetic analysis revealed that *A. pilosa* var. nepalensis and *A. pilosa* are closely related. Result of this study could provide genetic information for further research of *A. pilosa* var. nepalensis.

*Agrimonia pilosa* var. nepalensis (D. Don) Nakai is an herbaceous species of Rosaceae, and is widely distributed in Shannxi, Gansu, Yunan, and Zhenjiang province of China. It has ornamental and ecological values. Because of its good resistance and conservation capacity of soil and water, it is an excellent ground cover ornamental plant. Besides, given the medicinal value of its relative A. pilosa in Korea (Heo et al. 2020), this species may also have medicinal potential. However, the lack of genetic background seriously hinders its further research. Chloroplast genomes are relatively conservative, the genomes of higher plants are of great significance to study of molecular mechanism of photosynthesis, genetic improvement, taxonomic classification, and evolution history (Li et al. 2019; Daniell et al. 2021). Therefore, in this study, in order to provide genetic information for further studying of *A. pilosa* var. nepalensis, the complete chloroplast (cp) genome was determined based on the Illumina sequencing dataset.

Fresh leaves of *A. pilosa* var. nepalensisn (D. Don) were collected on July 28th, 2019 from the Xunyangba, Ankang, Shannxi province, China (108°32′54ʺE, 33°33′16ʺN, 1678 m H). The voucher specimen (Ap2020JulSH08) was deposited in the herbarium of Xinyang Agriculture and Forestry University. Total genomic DNA was isolated from the leaves by using modified CTAB method (Healey et al. 2014). DNA library construction and high-throughput sequencing on Illumina HiSeq X Ten platform were performed in Novogene Inc. (Beijing, China).

A total of 5.4 G raw reads were quality-trimmed using CLC Genomics Workbench v8 (CLC Bio, Denmark), resulted trimmed reads were then used for the cp genome assembly process with that of *Hagenia abyssinica* (GenBank KX008604) as a reference using software MITObim 1.7(Hahn et al. 2013) and ARC (Hunter et al. 2015). The cp genome of *A. pilosa* var. nepalensis was generated by a total of 133,489 individual reads at an average coverage of 129.9× using MITObim v1.7, it was then verified by three long cp contigs obtained by using ARC. Its annotation was completed using the program GENEIOUS R8 (Biomatters Ltd., Auckland, New Zealand) (Kearse et al. 2012) by comparing with the cp genome of *Hagenia abyssinica* mentioned above. The coding sequences, tRNAs and rRNAs were further confirmed and manually adjusted.

The complete chloroplast genome of *A. pilosa* var. nepalensis is openly available under accession number GWHAZPO00000000 in the Genome Warehouse Database of National Genomics Data Center, China National Center for Bioinformation/Beijing Institute of Genomics, Chinese Academy of Sciences (CNCB-NGDC Members and Partners 2021), and has been deposited into GenBank under the accession number MW387437. It is a circular molecule of 155,147 bp in length, contains 85 protein-coding genes, eight ribosomal RNA genes and 37 transfer RNA genes. Like most sequenced cp genomes, this genome contains a typical tetrad structure, including a large single copy (LSC) of 84,480 bp, a small single copy (SSC) of 18,737 bp, and two inverted repeat (IR) regions of 25,965 bp for each. Its overall GC content is 36.9%.

A total of 21 representative cp genomes from Rosaceae were selected and downloaded from NCBI, and 62 homologous protein coding sequences (CDSs) were then extracted, concatenated, and aligned using MAFFT v7.017 plugin (Katoh et al. 2002) for constructed a phylogenetic tree. The tree was constructed using MEGA 6.0 with a bootstrap value of 1000 (Tamura et al. 2013) based on the Maximum-Likelihood (ML) analysis with the Tamura–Nei model of the concatenated homologous CDSs with that of model plant species *Arabidopsis thaliana* as outgroup. Phylogenetic analysis revealed that *A. pilosa* var. nepalensis and *A. pilosa* are most closely related (Figure 1), both are clustered to *H. abyssinica* with high support, suggest that phylogenetic analysis based on homologous CDSs from the cp genomes is consistent with morphological classification.

**Figure 1.**
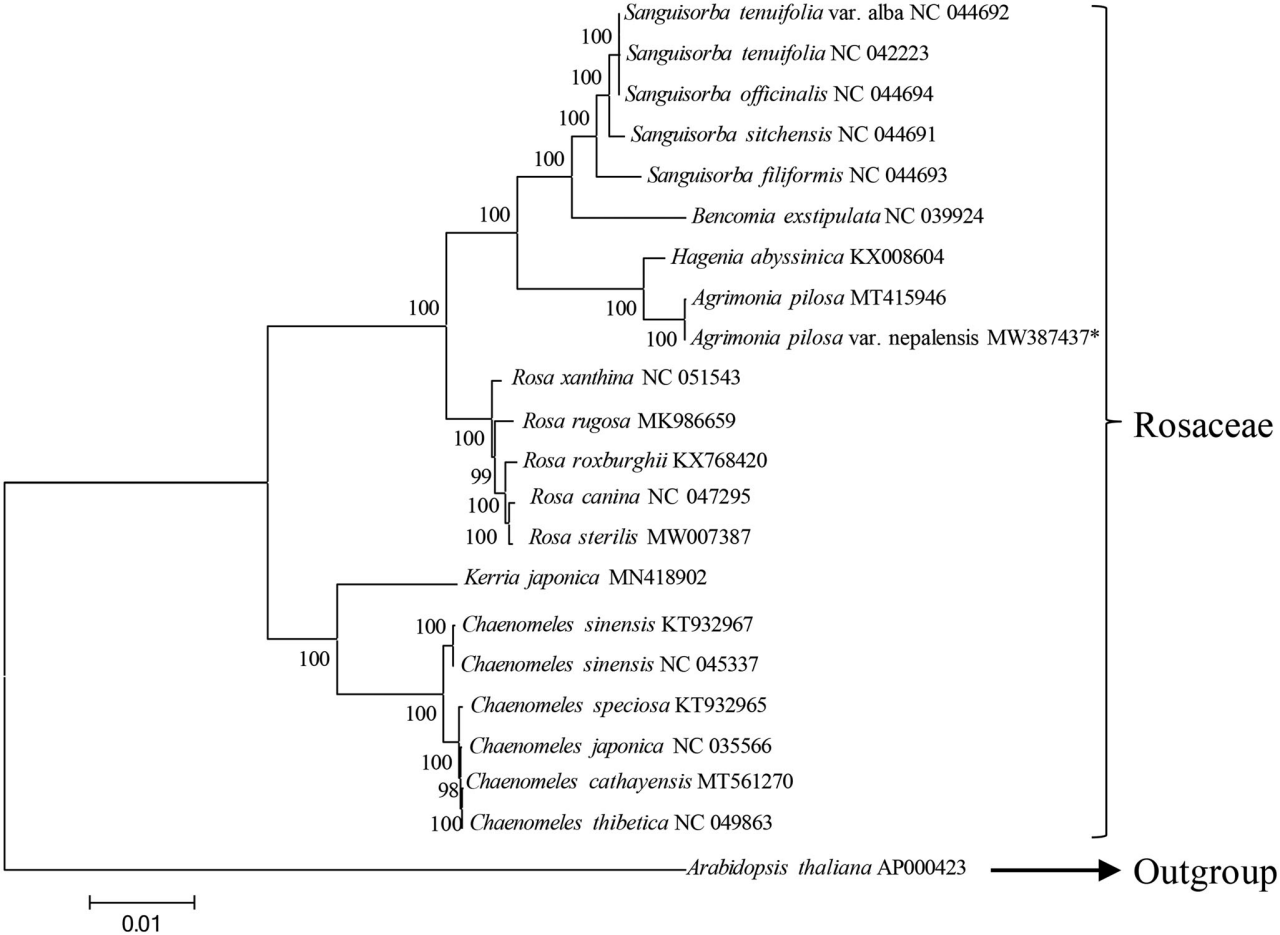
Phylogenetic tree based on 62 homologous protein coding sequences of 22 completed chloroplast genomes. The bootstrap value based on 1000 replicates is shown on each node. *This chloroplast genome is also openly available in NGDC under accession number GWHAZPO01000000 at https://bigd.big.ac.cn/gwh.

DnaSP v5 (Librado and Rozas 2009) was employed for DNA polymorphism analysis between *A. pilosa* var. nepalensis and its relative *A. pilosa* with 500 bp for window length and step size in the cpDNA genomic level, the result showed that single nucleotide polymorphisms (SNPs) were all found in single copy regions. A total of 21 SNPs were detected, including one SNP in SSC and 21 SNPs in LSC with nucleotide variability (*P*_i_) values ranged from 0 to 0.006, among them, relatively high variable loci (*P*_i_ ≥ 0.004) are located in *trnR*-*atpA*, *accD*-*psaI*, *rpoC2*-*rpoC1*, and intron of *petD*. Besides, insertion-deletions (indels) between the two cp genomes were all found in LSC, a total of 16 indels were detected, among them, indels of two nucleotides or greater in length are located in *trnR-atpA*, *accD-psaI*, *atpB-rbcL*, *petA-psbJ*, *rpl14-rpl16*, and intron of *trnL*. Given the relatively high polymorphic SNPs and indels exist between the two cp genomes, nine markers, including *trnR*-*atpA*, *accD*-*psaI*, *rpoC2*-*rpoC1*, *accD-psaI*, *atpB-rbcL*, *petA-psbJ*, *rpl14-rpl16*, intron of *petD*, and intron of *trnL*, could be selected as candidate polymorphic cp DNA markers for further research of identification of these two.

## Data Availability

The complete chloroplast genome constructed in study is openly available in the Genome Warehouse Database of National Genomics Data Center (NGDC), China National Center for Bioinformation/Beijing Institute of Genomics, Chinese Academy of Sciences, under accession number GWHAZPO01000000 at https://bigd.big.ac.cn/gwh, and also has been deposited into GenBank of NCBI under the accession number MW387437 at https://www.ncbi.nlm.nih.gov. The raw sequence data are openly available in the Genome Sequence Archive in NGDC of China under accession number CRA003774 at https://bigd.big.ac.cn/gsa. The associated BioProject and BioSample numbers in NGDC are PRJCA004281 and SAMC309461, respectively.
